# SVD-Based Mind-Wandering Prediction from Facial Videos in Online Learning

**DOI:** 10.3390/jimaging10050097

**Published:** 2024-04-24

**Authors:** Nguy Thi Lan Anh, Nguyen Gia Bach, Nguyen Thi Thanh Tu, Eiji Kamioka, Phan Xuan Tan

**Affiliations:** 1 School of Engineering Pedagogy, Hanoi University of Science and Technology, Hanoi 100000, Vietnam; anh.ntl202019@sis.hust.edu.vn (N.T.L.A.); tu.nguyenthithanh@hust.edu.vn (N.T.T.T.); 2 Graduate School of Engineering and Science, Shibaura Institute of Technology, Tokyo 135-8548, Japan; nb23505@shibaura-it.ac.jp (N.G.B.); kamioka@shibaura-it.ac.jp (E.K.)

**Keywords:** mind wandering, online learning, temporal eye signal, singular value eecomposition

## Abstract

This paper presents a novel approach to mind-wandering prediction in the context of webcam-based online learning. We implemented a Singular Value Decomposition (SVD)-based 1D temporal eye-signal extraction method, which relies solely on eye landmark detection and eliminates the need for gaze tracking or specialized hardware, then extract suitable features from the signals to train the prediction model. Our thorough experimental framework facilitates the evaluation of our approach alongside baseline models, particularly in the analysis of temporal eye signals and the prediction of attentional states. Notably, our SVD-based signal captures both subtle and major eye movements, including changes in the eye boundary and pupil, surpassing the limited capabilities of eye aspect ratio (EAR)-based signals. Our proposed model exhibits a 2% improvement in the overall Area Under the Receiver Operating Characteristics curve (AUROC) metric and 7% in the F1-score metric for ‘not-focus’ prediction, compared to the combination of EAR-based and computationally intensive gaze-based models used in the baseline study These contributions have potential implications for enhancing the field of attentional state prediction in online learning, offering a practical and effective solution to benefit educational experiences.

## 1. Introduction

Mind-wandering refers to the phenomenon where attention drifts away from the current task at hand, shifting to internally generated thoughts rather than being prompted by the external environment. This mental content is often described as unrelated to the ongoing task or independent of external stimuli, highlighting its detachment from perception and immediate actions [1,2]. Researchers have noted that mind-wandering has significant implications for education, as it is most noticeable when individuals are engaged in studying [3]. However, it is generally believed that mind-wandering mainly has negative effects on education, as it disrupts learners’ ability to concentrate.

With the increasing popularity of online education, learners now have access to a wide range of courses and programs, benefiting from the convenience, flexibility, affordability, self-paced learning, dynamic and engaging learning environments, and a global learning community. Nevertheless, online learning environments are more susceptible to mind-wandering compared to traditional classrooms [4], and this can be attributed to various factors such as reduced social interaction, increased cognitive load, higher potential for distractions, diminished problem-solving and critical thinking, challenges in time management, and the lack of physicality [5]. Consequently, it becomes crucial to capture attentional states and detect mind-wandering in the context of online learning.

Recent studies have utilized hardware-based methods to predict attentional states using various sensors [6]. These sensors include eye trackers, electroencephalography (EEG) sensors, electrodermal activity (EDA) sensors, and functional magnetic resonance imaging (fMRI) machines. For instance, the GazeTutor system [7], which utilizes eye trackers, has been able to identify students’ attentional states and provide dialogue to re-engage them and enhance learning outcomes. Another approach, called Attention-Aware Learning Technology (AALT) [8], uses eye trackers to predict mind-wandering and offer interventions such as asking questions, revisiting content, and calling students’ names.

However, these methods have some limitations. First, they require the use of specialized hardware. Second, their accuracy in predicting attentional states is relatively low. For example, the eye tracking-based AALT could only provide interventions in half of the total sessions with a low accuracy model, achieving a 0.51 F1 score [8]. Additionally, these methods have mainly been evaluated in controlled environments, and their performance in realistic settings remains unknown. Lastly, they often rely on education experts to label facial videos, which can be time-consuming and resource-intensive [9].

To tackle the limitations of hardware-based approaches, a recent paper about “Predicting Attention with Facial Expression” (PAFE) [6] interprets mind wandering from only webcam videos in online lectures with arbitrary settings (uncontrolled environment) and obtains more reliable prediction performance. Specifically, they construct an attentional state prediction model from their collected PAFE dataset, incorporating multiple physiology-related features such as eye aspect ratio (EAR), emotion, gaze, and head movement, extracted solely from video frames without support from specialized hardware. Their t-test findings reveal that EAR is the key indicator (*p* < 0.001) of mind-wandering. Nevertheless, a combination of the most important features in each feature category (EAR, gaze, emotion, head movement) is required to outperform a single-category model, such as a gaze-only baseline, as stated in their experiment results. However, combining these features requires separate eye landmark detection and resource-intensive gaze-tracking models, which hampers real-time attention prediction in online learning. To tackle this problem of facial-based attentional state prediction, our study proposes an alternative solution: using only eye landmarks to capture the whole eye region, which contains both EAR variations and gaze movements. Any subtle or high changes in this whole eye region from a facial video need to be well indicated by a 1D temporal signal, from which the features are obtained. An effective 1D temporal eye signal should (1) capture sufficient features to improve the prediction model of attentional states and (2) maintain a low computational cost during signal extraction.

This study aims to clarify the first goal, to effectively extract 1D temporal signal from a 2D image sequence in terms of predicting attentional states, which can be evaluated based on Accuracy, F1-score, and AUROC metrics. A set of 2D image sequences contains high-dimensional spatial and temporal information, and thus, a suitable feature dimensionality reduction method is needed. Prior studies have used Singular Value Decomposition (SVD) to achieve the temporal features extraction, either to capture scene background [10] or eye-blink signals [11]. In this study, we focus on isolating the eye region from facial video frames and applying SVD to generate 1D temporal eye movement signals based on higher-rank information from singular vectors. These signals capture all temporal changes in the eye region, encompassing eye boundary and pupil movements during different attentional states. The resulting eye movement signals are used to extract statistical and spectral features, creating custom datasets to train a classifier model.

This paper makes the following contributions:

- We implemented an SVD-based 1D temporal eye-signal extraction for attentional state prediction in webcam-based online learning, requiring only eye landmark detection, without gaze tracking or any specialized hardware support.
- We designed a thorough set of experiments pipeline for evaluation of our proposal with other baseline models in the context of analyzing and predicting attentional state.
- Our SVD-based 1D temporal signal can capture subtle or major movements of both eye boundary and eye pupil, whereas EAR-based 1D temporal signal can only reflect eye boundary variations, requiring additional gaze tracking to capture eye pupil variations.
- Our proposed SVD-based attentional state prediction model outperformed the combination of EAR-based and gaze-based models in state-of-the-art webcam-based mind-wandering prediction study [6] by 7% for F1-score in predicting ‘not-focus’, and 2% in the AUROC metric, indicating the degree of separability between “Focus” and “non-Focus” states for the prediction model.

## 2. Related Work

### 2.1. Specialized Hardware-Based MW Detection

The use of highly sophisticated optics and photonics devices for high accuracy of eye tracking has been a common trend in past research. The authors in [12] use Tobii 4C gaming eye-tracker with a 90 Hz sampling frequency, utilizing a reflection pattern of NIR (near-infrared) light for the recording of eye movements. The technology involves directing a light source toward the eye and using sensors to detect the reflection patterns of the light from the cornea and pupil. This method allows for the precise tracking of gaze points, fixation durations, and saccades, which are rapid eye movements between fixations. The study employs this technology to identify patterns indicative of mind-wandering, such as decreased fixation on relevant text areas or increased fixation dispersion, which may indicate a lack of focus. Another study [13] utilizes a screen-based Tobii Pro Spectrum eye-tracker with an even higher sampling rate of 300 Hz to analyze eye movement and oculomotor data as indicators of mind-wandering during video lectures. The specifics of eye-tracking technology include the use of high-resolution cameras that can capture the nuanced movements of the eye with extreme precision. This approach also encompasses the analysis of several eye movement metrics such as blink rate, saccade velocity, and fixation patterns based on gaze point, allowing researchers to determine where and how long a person is looking at specific points on a screen. Other studies also include the use of specialized optics hardware [14,15,16] but with less sampling rate and rely more on the biosensors such as EEG [5,6,10,11,15,16,17,18,19,20,21], EDA [5,20,22], or fMRI [21,23]. Despite the high precision of these specialized optics devices, they still require other unpleasant equipment, such as a head chin rest or head strap. This can be inconvenient for experimental use of testing subjects, leading to less accurate results, or for learners who use it for the long term. More recent advancements in eye-tracking technology, such as the wearable Neon eye-tracking glasses with a sampling rate of 200 Hz [24], enable more passive monitoring that might not affect the learner while maintaining good precision, and thus it is a potential solution for future mind-wandering-related studies. However, these mentioned eye trackers remain relatively expensive on the market and are thus not yet suitable in the context of online learning.

### 2.2. Facial Video-Based Mind-Wandering Detection

Trading off with the high precision of optics devices to maintain users’ learning process without affecting their experiences and with affordable price, the mind-wandering detection technologies begin to transition into webcam-based solutions, with sampling rates up to 60 Hz [25]. Overall, there is still a limited number of webcam-based methods for attention prediction during online courses without hardware support. Although a multimodal classifier integrating eye tracking and facial action has been recently implemented [6,26], partial eye-tracker equipment is still required for gaze tracking. Other webcam-only-based approaches are limited to controlled environments in labs, so they target limited focus on narrative film viewing [19,20] or show unreliable performance [21]. To tackle these limitations, a recent paper about “Predicting Attention with Facial Expression” (PAFE) [6] interprets mind wandering from only webcam videos in online lectures with arbitrary settings (uncontrolled environment) and obtains more reliable prediction performance. Specifically, they construct an attentional state prediction model from their collected PAFE dataset, incorporating multiple physiology-related features such as eye aspect ratio (EAR), emotion, gaze, and head movement, extracted solely from video frames without support from specialized hardware. Their t-test findings reveal that EAR is the key indicator (*p* < 0.001) of mind-wandering. Nevertheless, a combination of all facial-based features, including EAR, gaze, emotion, and head movement, is required to outperform a single-category model, such as a gaze-only baseline, which is frequently used in hardware-based approaches. However, combining these features requires separate eye landmark detection and resource-intensive gaze-tracking models, which hampers real-time attention prediction in online learning.

### 2.3. Feature Extraction

Given a set of 2D image sequences containing high-dimensional spatial and temporal information, a suitable feature dimensionality reduction method is needed to efficiently extract temporal information in the context of analyzing eye behavior. Feature dimensionality reduction can either be done by feature selection (keeping only a subset of features) or by feature extraction (generating a reduced number of new features from the original features). Since existing features (a set of pixels) in a 2D image sequence jointly combine both spatial and temporal information and we need to separate only the temporal features, the feature extract method is selected. The reduced number of new features after feature extraction can also be regarded as low-dimensional representations, from which the original features can be reconstructed using either linear or non-linear models. For the linear approach, prior studies have used Singular Value Decomposition (SVD) to achieve the temporal features extraction, either to capture scene background [10] or eye-blink signals [11]. For the non-linear models, presently, the autoencoders, a form of neural networks, have been commonly used to learn these low-dimensional representations for extracting temporal [27] or both spatiotemporal features from images [28] due to its ability to well preserve locality. A suitable feature extraction approach for our problem in this paper should be chosen based on the following criteria: interpretability, flexibility, and computational cost. Regarding interpretability, SVD provides a more interpretable decomposition of the data, where singular values directly represent the importance of features, from which we select the main one to represent an eye signal. Autoencoders are often treated as black boxes, and interpreting the hidden layer activations can be challenging. Regarding flexibility, SVD is a linear dimensionality reduction technique, while autoencoders can learn complex, non-linear relationships in the data. This allows autoencoders to potentially capture more nuanced low-dimensional representations. Regarding the computational cost, SVD is a well-established algorithm with a guaranteed closed-form solution, whereas training autoencoders requires significant computational resources. Given the context of predicting attentional states from an interpretable eye signal in online learning with real-time capability, the SVD-based feature extraction approach is selected in our paper.

## 3. Problem Statement

**Figure 1:**
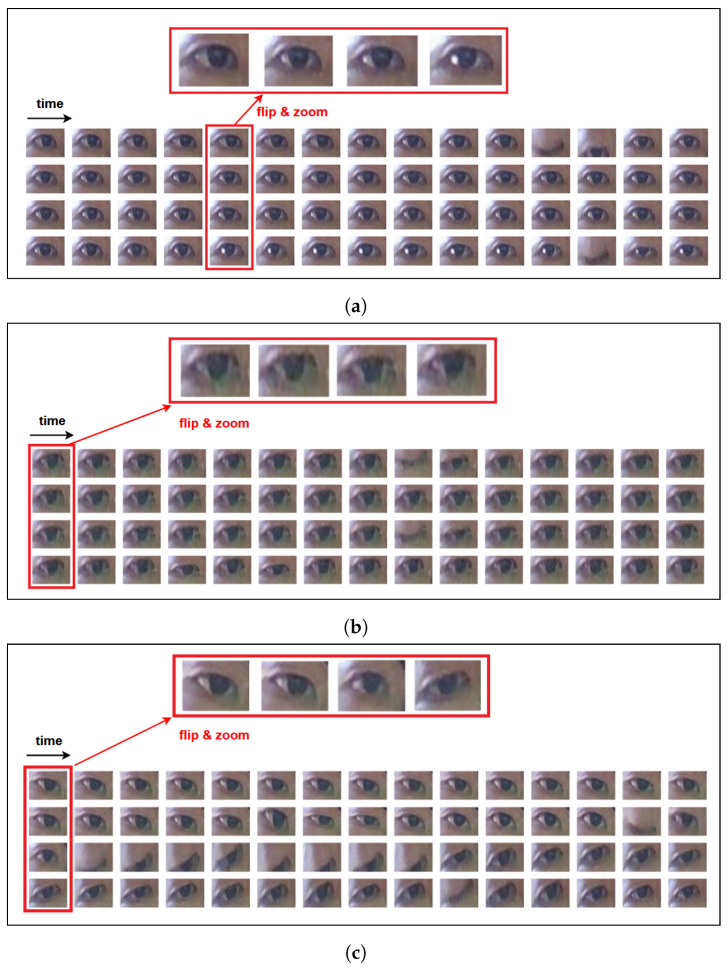
Example of the eye-image sequence in “Focus” and “non-Focus” states from PAFE dataset [6]. The eyes pictures are ordered from left to right and top to bottom. The example sequences are sampled at 15 FPS for ease of illustration. (**a**) “Focus” state: eye boundary mostly remains stationary, while the eye pupil actively moves horizontally. (**b**) “non-Focus” state: Inactivity in eye pupil (long fixation). (**c**) “non-Focus” state: Inactivity in eye pupil (long fixation) or high activity of eye boundary and facial/head movements, leading to variances in orientation.

The work proposed in PAFE [6] has shown that eye-related behaviors like eye aspect ratio (EAR) features are the most significant indications of mind wandering (“non-Focus” state). However, from our observations of both “Focus” and “non-Focus” states in the PAFE dataset and previous findings in other eye gaze-based studies [23,26,29,30,31], another indication of attentional state could be embedded in the movement of pupils, which has not been reflected in the EARs, revealing only the ratio of eye boundary. For instance, a high number of “Focus” samples indicate high pupil movement activity, whereas the eye boundary remains stationary, except in the cases of blinks. In contrast, other “non-Focus” samples contain either inactivity in the eye pupil and/or high activity of both eye regions (pupil and eye boundary) and other facial or head movements. Figure 1 illustrates examples of left eye-image sequences in “Focus” and “non-Focus” states.

As a result, in order to obtain robust performance in the prediction of attentional state, both the EAR-based features and gaze-based features are combined in [6] to train the learning model. However, this requires separate eye landmark detection and heavy-computation gaze detection models, leading to more resource consumption and hindering real-time attentional state prediction applications in online learning. Therefore, an alternative solution is needed to use solely eye landmarks and capture the whole eye region, which contains spatial and temporal information of both EAR variations and gaze movements, producing significant features of the combined categories while maintaining a good prediction model.

Since only temporal changes indicate attentional states, the main challenge is to extract 1D temporal signal information effectively from a spatiotemporal 2D eye-image sequence cropped out from the facial video frames. To tackle this problem, this paper is inspired by recent Singular Value Decomposition (SVD) -based studies, extracting spatial or temporal information from a 2D video sequence. This approach allows the capture of either scene background based on the low rank of left/right singular vectors [10] or eye-blink signals based on higher-rank information representing temporal changes [11].

## 4. Proposed Methodology

**Figure 2:**
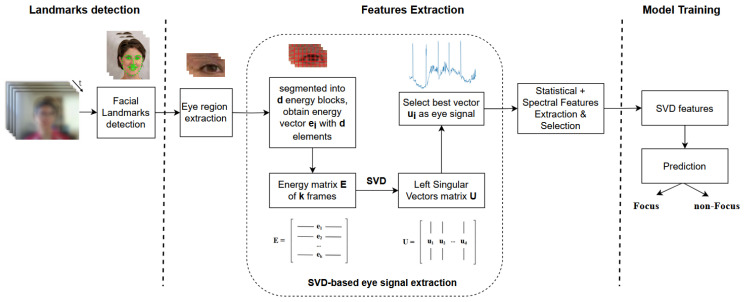
Overview of the proposed SVD-based approach.

An overview of our proposed SVD-based mind-wandering detection is depicted in Figure 2, consisting of three main steps: (1) extracting raw frames from an existing video dataset and detecting facial landmarks, (2) locating eye region and extracting SVD-based eye-signal features, and (3) training machine-learning models on datasets of extracted features. This section focuses on Step 2, which contains our main contribution, and implementation details on Steps 1 and 3 can be found in the next Experiment Section 5.

Regarding Step 2, given the extracted facial landmarks, the approach isolates only the eye landmarks to capture the eye regions. Then, SVD is utilized to extract eye movements signal, capturing all temporal changes in the whole eye region, which include eye boundary movements and pupil movements during attentional states.

Specifically, the SVD-based eye-signal extraction is carried out as follows. Given a cropped eye-image sequence $J=\left\{I_{1},I_{2},\cdots ,I_{k}\right\}\in R^{m\times n\times k}$, with m,n,k as image height, width, and the number of frames, each image $I_{i}$ is divided into *d* blocks. Each block’s pixel energy is computed as the sum of the square intensities of all pixels within that block. Thus, each image $I_{i}$ corresponds to a d-dimensional energy vector $e_{i}\in R^{1\times d}$, containing *d* elements of block energy values. Subsequently, image sequence *J* with *k* frames corresponds to an energy matrix $E=\left\{e_{1};e_{2};\cdots ;e_{k}\right\}\in R^{k\times d}$, where each row is the d-dimensional energy vector per image. The energy matrix *E* contains joint spatial and temporal information of the image sequence. Every row in *E* represents the spatial correlations between adjacent pixel blocks within each video frame, whereas every column in *E* indicates temporal correlations of the same pixel block between adjacent video frames. Previous study [10] has shown that the temporal information can be separately extracted from E using SVD, decomposing matrix *E* as follows:

(1) $$U^{T}EV=\Sigma =\mathrm{diag}\left(\sigma_{1},\sigma_{2},\ldots ,\sigma_{p}\right)\in R^{k\times d}$$

in which p=min(k,d) and $\sigma_{1}\geq \sigma_{2}\geq \ldots \geq \sigma_{p}\geq 0$, $U\in R^{k\times k}$ and $V\in R^{d\times d}$ are the left and right singular vectors, respectively.

A reduced-size matrix *U* is commonly used [10], where the number of rows is reduced to *d*, resulting in $U=\left\{u_{1},u_{2},\ldots ,u_{d}\right\}\in R^{k\times d}$. According to [10], the same temporal information of the original spatiotemporal energy matrix *E* is compacted in the left singular vector matrix *U*, and thus its structure should be further investigated. As in signal processing, the projection of *E* into the first left singular vector $u_{1}$ of *U* uncovers the low-rank details present in *E*. Meanwhile, the projections into the remaining singular vectors $u_{2},\ldots ,u_{d}$ indicate sparse or high-frequency information of temporal changes. This means any subtle or major changes in temporal dimension should be reflected distinctly in one of the higher-rank singular vectors $u_{2},\ldots ,u_{d}$ [11], and thus it is necessary to select the vector that best represents the eye change signal.

The left singular vectors first need to be preprocessed. Each vector contains both positive and negative values within the range of −1 to 1. To enhance the representation and analysis of the estimated eye signal, we scale the values in the selected vectors to fit within the range of [0 1]. These scaled vectors are then subject to temporal processing via a moving average filter to minimize outliers and noises. Subsequently, the most suitable vector to represent changes in the eye signal is selected based on its frequency characteristics. The higher the changes in the temporal dimension, the higher the coefficient amplitudes in the frequency domain in its high-frequency components range. Hence, each vector is transformed into the frequency domain using Fast Fourier Transform (FFT) [32], and the best candidate is selected as the vector with the highest frequency amplitude within a predefined interval. Afterward, the obtained best left singular vector representing the 1D temporal eye change signal is later used to extract statistical and spectral features in the experiments and establish custom datasets, which are then used to train the classifier model.

## 5. Experiments

This section first introduces an overview of the experiment pipelines, including both baseline methods and the proposed SVD-based approach, then provides details on the evaluated dataset, implementation details, and each component in the features extraction step. Subsequently, analysis and evaluation are given on the extracted temporal eye signals and the performance of attentional state prediction models based on the features extracted from those signals. Our code implementation can be found in the following repository: https://github.com/bachzz/MW-SVD (accessed on 1 April 2024).

### 5.1. Overview Experiment

**Figure 3:**
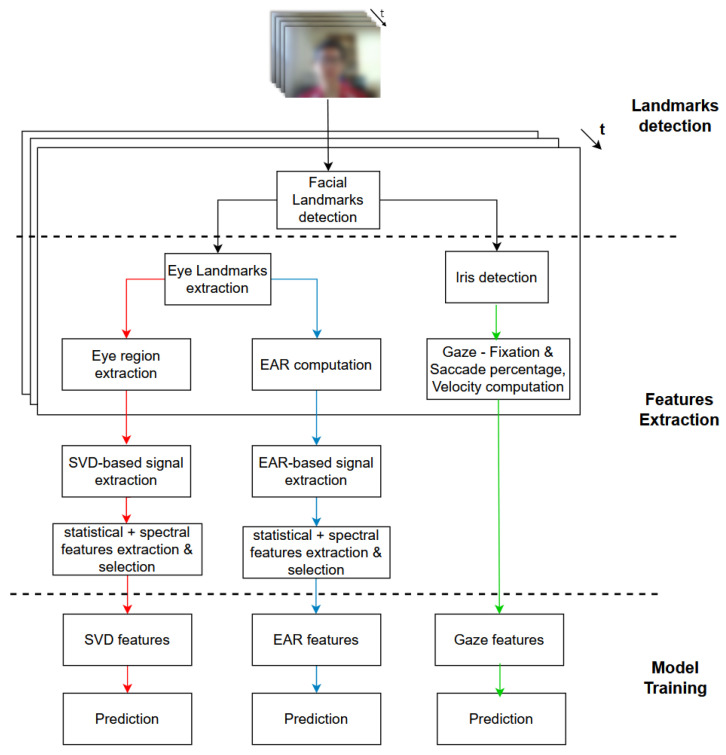
Overview of experimental scenarios, including the proposed SVD-based approach (red), and the baseline methods: EAR-based [6] (blue), Gaze-based [26,29] (green).

The overview pipelines in Figure 3 illustrate three main steps for the experiment procedures of each scenario, extending the steps of our proposed SVD-based method in Figure 2 to the other baseline methods. In Step (1), we investigated the existing recorded videos of participants during online learning from the original PAFE dataset [6], in which the authors evaluated their proposed EAR-based and gaze-based features. The details on this dataset can be found in the next Section 5.2. In Step (2), feature extraction is also carried out for EAR-based signal and gaze behavior, in addition to SVD-based signal. In Step (3), the learning models are trained on datasets of extracted features for each corresponding approach. The implementation details of each step are illustrated in Section 5.7.

### 5.2. Dataset

**Figure 4:**
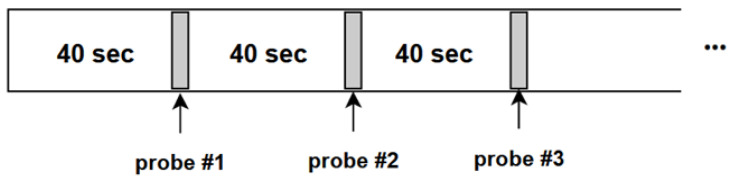
Periodic Probing used in PAFE experiment [6].

As described in [6], during the dataset recording process of the PAFE experiment, the participants were watching the same lecture video, “AI For Everyone” by Andrew Ng, at their preferred date and alone in their room, without disturbance from smartphones, or any apps alerts, notifications. The authors implemented a periodic probing method (by a ding sound every 40 s, as shown in Figure 4) for the participants to report their attentional states. To reduce the distraction effect of probing sound, they utilized only the last 20 seconds in every 40-second interval for later processing steps such as feature extraction. This is supported by previous findings that our thoughts are shifted from 5 to 30 s [33]. For every probing time window, given 30 FPS in their experiment setup, the investigated 20-second video produces a sequence of 600 frames for further processing, with a single label (i.e., “Focus”, “non-Focus”, “Skip”). Our experiment extracts only “Focus” and “non-Focus” sequences, excluding “Skip” sequences, in which the participants could not immediately decide the response. Eventually, we obtained sequences of 5 participants, each participant containing about 80 labeled sequences of 600 frames, and each frame has a resolution of 640 × 480. Each sequence of 600 frames also corresponds to an eye signal. As a result, the total number of data samples or eye signals is around 400, and the proportion of ’Focus’ and ’Non-Focus’ labels after preprocessing is about 5 to 1, respectively.

### 5.3. Implementation Details

This section provides the experimental details of each component described in Figure 3. In Step (1), facial landmark detection was carried out using HRNet [34]. In Step (2), from the facial landmarks, left and right eye landmarks were extracted from points 60 to 67 and 68 to 75, respectively, which were used in the EAR-based and SVD-based approaches. In our proposed SVD-based solution, the eye region was then cropped surrounding the target eye landmarks by 25% in all directions. Then, the cropped eye images were resized into 96 × 128 pixels. For the gaze-based method, iris detection was carried out using MediaPipe Facemesh [35] to locate the coordinates of the iris. The extracted eye region and landmarks were then used to produce 1D temporal eye signals for EAR-based and SVD-based approaches. Since these signals are 1D time series data, their features should be extracted with a suitable tool, for which we used the Time Series Feature Extraction Library (TSFEL) [36]. On the other hand, the detected iris locations over a sequence were used to compute gaze-related features: horizontal fixation percentage, horizontal saccade percentage, and horizontal saccade velocity, as single values representing the whole sequence, instead of 1D time series data [26,29]. Afterward, the extracted features of each approach were further selected based on their importance (*t*-test) to create different datasets for prediction model training. In Step (3), following the same model learning strategy of the baseline study [6], a traditional machine-learning technique, XGBoost, and a simple Deep Neural Network (DNN) were utilized to learn from the generated datasets. The XGBoost model was set up with 1000 boosting rounds and an “AUC” evaluation metric for validation data. Regarding the DNN architecture, since the input features are structured numerical data, a simple Multi-Layer Perceptron (MLP) network was utilized in the original experiment proposed in [6]. Following their design, we implemented the same MLP model consisting of two layers: the first one contains 12 nodes with ReLU activation, and the second one contains 1 output node with Sigmoid activation, along with binary cross-entropy loss and Adam optimizer.

### 5.4. Features Extraction

#### 5.4.1. Proposed SVD-Based Approach

As discussed in Section 4, the obtained best left singular vector represents the 1D temporal eye change signal. From the given signal in the time series domain, this paper extracts SVD-based features from their corresponding statistical domain and spectral domain, utilizing the Time Series Feature Extraction Library (TSFEL) [36]. An overview of the investigated features description is given in Table 1.

Since SVD-based eye temporal signals contain both changes in eye aspect ratio and pupil movement, the signal patterns seem to be “noisy” with peaks of blinking. However, the “noisy” fluctuations actually indicate pupil movements, but they are excluded by statistical feature extraction. On the other hand, noisy data features are better represented and handled in the spectral domain of the signal [37]. Thus, spectral feature extraction is more suitable for SVD-based eye signals.

#### 5.4.2. Baseline Methods

(a) EAR-based approach

**Figure 5:**
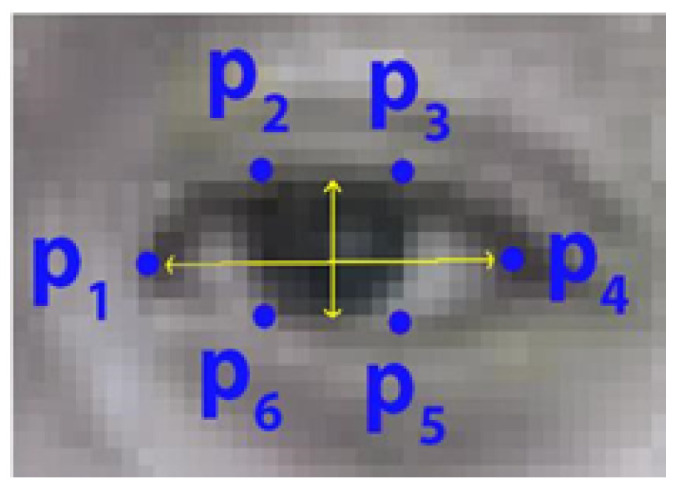
An example of eye landmarks, representing ratio of eye height over width.

As illustrated in Figure 3, from the extracted eye landmarks of each frame, the eye aspect ratio (EAR) representing eye height over eye width is computed using Equation (1) [34], in which the eye landmarks are denoted from p1 to p6, as shown in Figure 5. The ratio is scale-invariant, which is not affected by the varying distances from the eye to the camera. Furthermore, the facial landmark detection system, along with the Perspective-n-Point (PnP) algorithm, provides translation invariance and rotation invariance [6]. Thus, EAR should be robust to potential variances, provided that the eye is detectable. Additionally, following the procedure proposed in [6], the EAR values are then scaled with median and median absolute deviation (MAD) of the initial 20 s during data collection, assuming most participants are fully focused at the beginning, in order to achieve per-participant invariance. This scaling allows the strategy to be applicable to any session. Furthermore, missing EAR values as a result of failed facial landmark detection are linearly interpolated.

(2) $$\mathrm{EAR}=\frac{∥p_{2}-p_{6}∥\;+\;∥p_{3}-p_{5}∥}{2∥p_{1}-p_{4}∥}$$

Repeating the process for a sequence of frames, we obtain a time series data of EAR values, resulting in an eye signal. From the obtained time series data, their features also need to be extracted in the corresponding statistical domain and spectral domain, utilizing TSFEL [36]. The EAR-based signal is frequently used in blink-based partial drowsiness detection, which has been reported as a strong indication of attentional states [6,38,39]. Thus, the EAR-based features extracted from the signal should focus on clear blinking factors, reducing the short-term effects of noisy data. Since EAR reflects only movements of eye boundary, the noisy data (i.e., partial blinks) does not include useful information such as eye pupil movements, unlike SVD-based signal. As a result, statistical features excluding outliers (i.e., partially closing eyes) are suitable to be extracted from the EAR-based eye signal. Nevertheless, its features in the spectral domain were also extracted for comparison with the SVD-based approach.

(b) Gaze-based approach

There are three major components of gaze movements during online learning [6] and computerized reading [10]: speed, fixations, and saccades. Although our eyes generally seem to be gliding smoothly across the page of text as we read the slide content, in reality, they make a series of rapid movements (called saccades, which move the eyes from one place to another in the screen) separated by pauses (called fixations, which typically last roughly 200–250 ms during focus, and longer during mind wandering) [40]. These indications can be obtained solely based on iris tracking [41].

For each frame, the coordinate of the iris is first computed within the region of detected facial landmarks. Repeating the computation for a sequence of frames, we obtain a time series data of iris coordinates. Based on the coordinate information, the horizontal velocity of eye movement, the fixation percentage, and the horizontal saccade percentage are calculated. Each frame is marked as “saccade” if the iris’s horizontal position in the current frame is different from the previous frame by a threshold of pixels (in the experiment, we use a threshold of 1 pixel). Each frame is marked as “fixation” if the iris’s horizontal position does not change with respect to the previous frame. Then, the horizontal fixation percentage and the horizontal saccade percentage are calculated as the number of “fixation” and “saccade” frames, respectively, over the total number of frames in the eyes sequence window. The horizontal velocity of pupil movement is calculated during continuous “saccade” frames as the total pixel movement over the number of “saccade” frames.

It should be noted that in this experiment, due to a lack of hardware resources and lack of gaze-calibration files from the computer monitors of participants in the PAFE dataset, we could not use the same heavy-computation gaze estimation approach from the original baseline attentional state prediction work [6]. In their work, the authors utilized the Few-Shot Adaptive Gaze Estimation (FAZE) [42] method for gaze tracking and extracting gaze features. Despite the real-time ability for inference with a live webcam, FAZE requires heavy-computation training: 8x GPUs, each one is Tesla V100 GPU with 32 GB memory [43]; meanwhile, the inference process still requires multi-GPU support [44]. Additionally, on inference with new subjects that have not been learned, FAZE requires a gaze-calibration process (approximately 10 s) and further training [42]. On the other hand, the proposed SVD-based approach is mainly based on signal processing; hence, it requires no training or intensive memory, and no calibration process for new subjects is needed. Therefore, as an alternative approach, we utilized light-computation MediaPipe Facemesh [35] to detect iris coordinates in a gaze-based baseline, which is adequate to compute the above gaze-based features: horizontal fixation percentage, horizontal saccade percentage, horizontal pupil velocity.

### 5.5. Analysis of Temporal Eye Signals on Eye Activities

**Figure 6:**
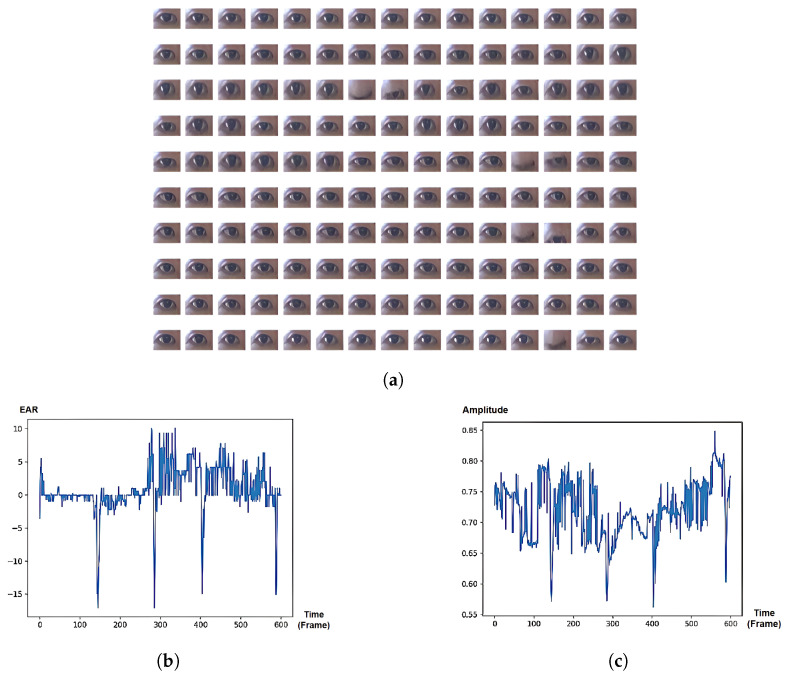
“Focus” state: eye boundary mostly remains stationary, while the eye pupil actively moves horizontally. (**a**) Eye images sequence from PAFE dataset [6], consisting of 4 blinks and active pupil movements. The eyes pictures are ordered from left to right and top to bottom. (**b**) EAR-based eye signal, mainly capturing 4 blinks. (**c**) SVD-based eye signal, capturing both 4 blinks and pupil movement.

The experiment investigates the three most frequent cases of attentional states from the PAFE dataset. Regarding the eye sequences labeled as ‘Focus’ (Figure 6a), the eye boundary mostly remains stationary, while the eye pupil actively moves horizontally. It can be seen that both EAR-based and SVD-based eye signals can capture major changes in eye movement, such as blinks or partial blinks; however, the temporal changes in pupil during gaze movement can only be captured using our proposed SVD-based approach, excluding the need for gaze-tracking system.

**Figure 7:**
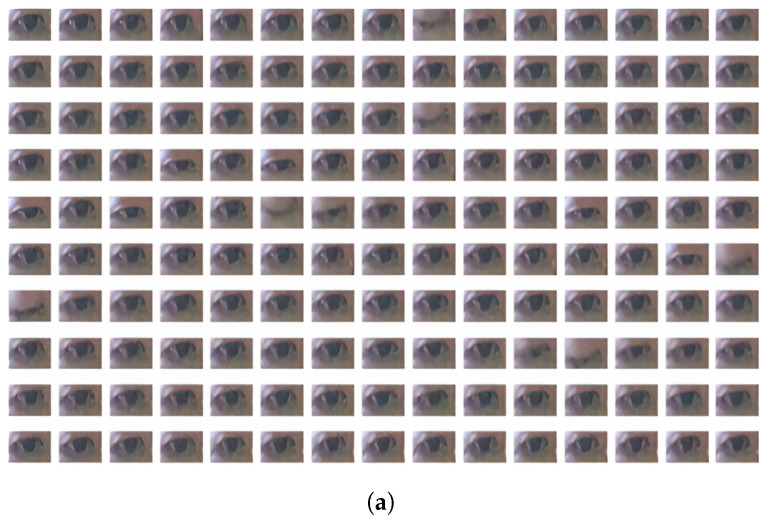

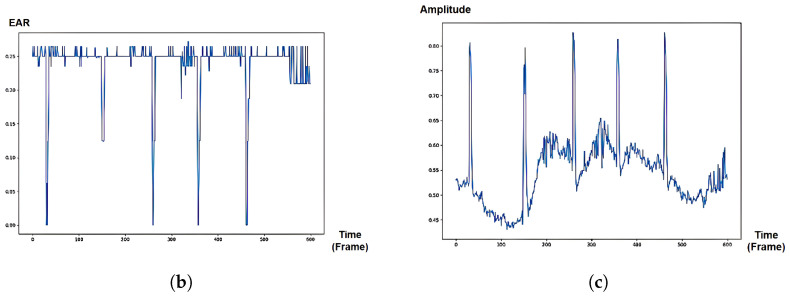
“Non-Focus” state: Inactivity in eye pupil (long fixation). (**a**) Eye mages sequence from PAFE dataset [6], consisting of 5 blinks and low activity in pupil movement. The eyes pictures are ordered from left to right and top to bottom. (**b**) EAR-based eye signal, mainly capturing 5 blinks. (**c**) SVD-based eye signal, capturing 5 blinks and low pupil activity.

**Figure 8:**
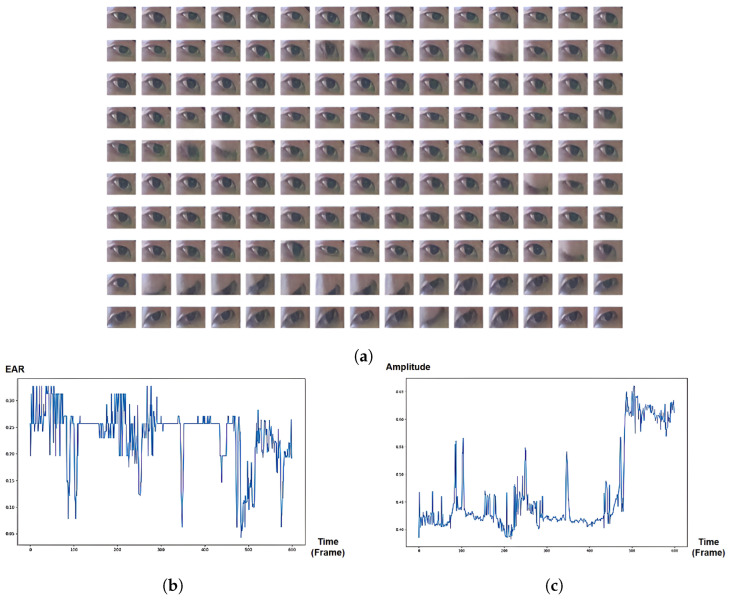
“Non-Focus” state: Inactivity in eye pupil (long fixation). (**a**) Eye-Image sequence from PAFE dataset [6], consisting of blinks, low pupil activity, and rapid translational/rotational changes caused by head movement. The eyes pictures are ordered from left to right and top to bottom. (**b**) EAR-based eye signal, mainly capturing blinks due to translation invariance and rotation invariance. (**c**) SVD-based eye signal, capturing blinks, low pupil activity, translational and rotational changes.

Regarding the eye sequences labeled as ‘non-Focus’, there is usually either inactivity in the eye pupil, causing long fixation (Figure 7a) or high activity of both eye boundary and facial/head movements (Figure 8a), leading to variances in eye orientation. Long fixation leads to low activity in pupil movement, which is also reflected in SVD-based eye signals. On the other hand, rapid translational/rotational eye changes caused by head movement are indicated with more significance in SVD-based than EAR-based signals because the eye aspect ratio is translation invariance and rotation invariance [6]. Therefore, our proposed SVD-based eye signal can capture more features of both “Focus” and “non-Focus” attentional states.

### 5.6. Features Selection and Generated Datasets

As discussed in Section 5.4, TSFEL [36] was used to extract both statistical and spectral features (as listed in Table 1) from the EAR-based and SVD-based eye temporal signal, whereas the Gaze-based approach utilized independent features: horizontal saccade velocity, horizontal fixation percentage, horizontal saccade percentage.

Following the procedure in [6], we performed a feature-elimination process based on the p-value technique. The *p*-value results used for feature selection were calculated based on the null hypothesis, indicating there is no relationship between a feature (predictor variable) and the target label (response variable). The lower the *p*-value, the stronger the evidence against the null hypothesis, and the more significant the feature is to the target label. Specifically, given a dataset with a set of features, we utilized the Ordinary Least Squares (OLS) module from the “statsmodels” library [45] in Python to estimate the parameters of a linear regression model. It internally calculates various statistics, including *p*-values. The *p*-values associated with the coefficients of the model are typically calculated based on the assumption of normally distributed errors for the coefficients. After fitting the model, OLS computes the standard errors for each estimated coefficient. These standard errors represent the uncertainty or variability in the estimated coefficients. For each coefficient, the t-statistic is calculated by dividing the estimated coefficient by its standard error. The t-statistic measures the number of standard deviations the coefficient estimate is away from zero. OLS uses these values along with the degrees of freedom to compute the p-values. The *p*-value associated with each coefficient tests the null hypothesis that the coefficient is equal to zero (i.e., there is no relationship between the predictor variable and the response variable). The lower the *p*-value, the stronger the evidence against the null hypothesis. In our experiment, we utilized threshold *p*-value = 0.05 to retain only important features.

Table 2 shows the statistics of only significant features (*p*-value ≤ 0.05) obtained after performing the t-test for the three approaches. It can be seen that the statistical features of EAR-based and spectral features of SVD-based methods share a high number of features with strong significance (*p*-value < 0.001). This corresponds well to the findings of previous studies [6,38,39] about using statistical features of EAR variations (excluding outliers) for blink-based detection of partial drowsiness, which is a strong indication of attentional states. On the other hand, since an SVD-based signal contains variations in eye aspect ratio and pupil movements, the features of its noisy data are better shown and extracted in the spectral domain, leading to higher significance than SVD-based statistical features in the temporal domain. Lastly, the table also showed that only the horizontal saccade percentage feature is retained for the gaze-based approach for having high significance.

As a result, we created 7 different datasets (5 baseline datasets and 2 proposed SVD-based datasets) to evaluate our proposed SVD-based mind-wandering detection during online learning. The utilized datasets are described in Table 3.

### 5.7. Evaluation Metrics

F1 score is often utilized over Accuracy for imbalanced dataset evaluation because it combines Precision and Recall. Accuracy can be misinterpreted when there is a skewed class distribution since it may be high even when the classifier performs poorly on the minority class. Precision is about how many of the positively predicted samples are actually positive, but it does not capture the full classifier’s performance by neglecting negative samples. Recall is about how many of the actual positive samples are predicted correctly, but it may be influenced by the class imbalance, causing inflated values. In contrast, the F1 score combines Precision and Recall into a single measure and balances the two affected metrics, where the relative individual contributions are equal.

AUROC (Area Under the Receiver Operating Characteristic Curve) measures the ability of the model to distinguish between the classes by telling how well the classifier can rank a random positive sample higher than a random negative sample [46]. AUROC can reflect the overall performance of the classifier across different threshold levels. It is less sensitive to class imbalance [47] because it focuses on the model’s ability to rank samples rather than directly on the absolute numbers of true positives and false positives.

Overall, the F1 score is suitable when dealing with an imbalanced dataset and when it is more important to predict the minority (positive) class accurately (high recall) while ensuring the predicted sample is indeed the minority class (high precision). On the other hand, if the problem concerns the overall classifier performance to separate both classes while minimizing misclassifications, AUROC might be the more suitable metric [46]. In the context of predicting attentional states with our currently limited and imbalanced dataset, both F1 and AUROC are necessary for evaluation since we equally care about both ’Focus’ and ’Non-Focus’ classes and their separability, in addition to balancing Precision and Recall when influenced by the imbalanced dataset. Future work may also investigate the use of data augmentation techniques to deal with the imbalanced learning problem.

### 5.8. Prediction Model Results and Discussion

The 7 generated datasets were used to train and evaluate 5 baseline models, and 2 proposed SVD-based models. Each model was utilized with either XGBoost or DNN for the datasets of the 20-second probing window. Since the datasets are imbalanced, consisting of approximately 5× labels of “Focus” than “non-Focus”, we implemented stratified 5-fold cross-validation and random undersampling for the training data in order to ensure the training and test sets with the same proportion in each fold as the original dataset and to reduce the imbalance effect.

Table 4 provides the experimental results. Overall, the XGBoost model outperforms the DNN models. The poor AUROC results of DNN models are likely caused by either insufficient training data (few participants), making the model mainly predict a single label and leading to results around 0.5, or the model learned from highly imbalanced labels, or the model choice issue (i.e., the selected features may have complex relationships that a simple model cannot capture). Thus, the use of DNN should be investigated further in our future work. Regarding the comparison between our proposal and baseline models, XGBoost model training on our proposed SVD-spectral features shows the highest AUROC performance (AUROC = 0.57), representing the highest capability separating the classification of “Focus” and “non-Focus”. Our proposed model outperforms the original EAR-stats baseline (AUROC = 0.53), the gaze-based baseline (AUROC = 0.54), and the combination of both baselines features EAR-spectral + Gaze (AUROC = 0.56). This indicates that the SVD-based eye signal can capture both features of EAR-based and Gaze-based methods, and the SVD-based features can provide better classification performance in the prediction of attentional states. Although the EAR-spectral+Gaze baseline slightly outperforms the proposed SVD-spectral by 1% in the accuracy of “Focus” predictions, the proposal still has the highest F1-score (=0.3) in “Not-Focus” predictions. The higher accuracy is likely due to the baseline model having a higher chance of predicting “Focus” for datasets with a large proportion of “Focus” labels. Additionally, the SVD-spectral model also outperforms the SVD-statistical model (AUROC = 0.52), aligning well with our assumption that the noisy features of both eye aspect ratio and gaze movement are better captured and exploited in the spectral domain.

## 6. Conclusions

In conclusion, our study presents a novel approach for predicting attentional states in webcam-based online learning environments. By leveraging Singular Value Decomposition (SVD) to extract 1D temporal eye signals from solely detected eye landmarks, our method offers a lightweight and accessible solution that eliminates the need for gaze tracking or specialized hardware. Through a rigorously designed experimental pipeline, we demonstrated the effectiveness of our proposal by comparing it against baseline models in the context of attentional state prediction. Importantly, our SVD-based eye signal exhibits the ability to capture subtle and major movements of both eye boundary and eye pupil, distinguishing it from existing EAR-based eye signals that primarily focus on eye boundary variations, which require additional gaze tracking to capture eye pupil variations. The experiment results demonstrate that our SVD-based attentional state model outperforms the baseline combination of EAR-based and gaze-based models by 2% and 7% in the AUROC and F1 metrics, indicating its ability to separate well between "Focus" and "non-Focus" states while better balancing between precision and recall, given a sequence of facial video frames. This highlights its potential for enhancing attentional state prediction in online learning scenarios. These contributions hold promise for enhancing the development of adaptive learning systems, allowing for the intervention of mind-wandering once detected and paving the way for improved online learning experiences. In future work, we intend to assess the real-time ability of the proposed system, gather more online learning datasets, and improve the performance of deep learning-based methods.

## Figures and Tables

**Table 1:** Overview of statistical and spectral features from the time series feature.

Features		Description
Statistical	Absolute energy	Computes the absolute energy of the signal
	Entropy	Computes the entropy of the signal using the Shannon Entropy
	Interquartile range	Computes interquartile range (Q3-Q1) of the signal
	Max	Computes the maximum value of the signal
	Min	Computes the minimum value of the signal
	Mean	Computes the mean value of the signal
	Mean absolute deviation	Computes mean absolute deviation of the signal
	Median	Computes median value of the signal
	Median absolute deviation	Computes median absolute deviation of the signal
	Standard deviation	Computes standard deviation of the signal
	Variance	Computes variance of the signal
	Peak to peak distance	Computes peak to peak distance of the signal
	Root mean square	Computes root mean square of the signal
	Kurtosis	Computes kurtosis of the signal
	Skewness	Computes skewness of the signal
Spectral	Max power spectrum	Computes maximum power spectrum density of the signal after Fast Fourier Transform (FFT)
	Maximum frequency	Returns the frequency with 95% of the Cumulative sum of the magnitude after FFT
	Median frequency	Returns the frequency with 50% of the Cumulative sum of the magnitude after FFT
	Power bandwidth	Computes power spectrum density bandwidth of the signal after FFT
	Fundamental frequency	Finds the lowest frequency of the signal after FFT
	Spectral centroid	Computes the barycenter of the spectrum after FFT
	Spectral decrease	Computes the amount of decreasing of the spectra amplitude after FFT
	Spectral distance	Compute spectral distance between Cumulative sum of the magnitude after FFT and its linear regression
	Spectral entropy	Compute Spectral entropy of the spectrum after FFT
	Spectral kurtosis	Computes the flatness of a distribution around its mean value in the spectrum after FFT
Statistical	Spectral skewness	Computes the asymmetry of a distribution around its mean value in the spectrum after FFT
	Spectral slope	Computes the spectral slope, obtained by linear regression of the spectral amplitude after FFT
	Spectral spread	Computes the spread of the spectrum around its mean value after FFT
	Spectral variation	Computes the amount of variation of the spectrum along time after FFT
	Wavelet energy	Computes Continuous Wavelet Transform (CWT) energy of each wavelet scale
	Wavelet entropy	Computes CWT entropy of the signal
	Wavelet variance	Computes CWT variance value of each wavelet scale.

**Table 2:** Statistics of only important features for EAR-based, Gaze-based, and SVD-based methods. The number of stars indicates the significance level of the feature.

Method	Domain	Features	Focused		Not-Focused		*p*-Value
			**Mean**	**SD**	**Mean**	**SD**	
EAR	statistical	Absolute energy	2027.00	920.69	2302.74	930.27	<0.001 ***
		Entropy	0.50	0.18	0.44	0.16	<0.001 ***
		Kurtosis	5.08	21.76	6.37	30.10	0.050 *
		Mean absolute deviation	0.23	0.47	0.25	0.47	0.001 **
		Root mean square	0.89	1.60	1.01	1.68	<0.001 ***
		Skewness	−0.88	1.70	−0.52	2.15	0.006 **
		Standard deviation	0.29	0.59	0.35	0.64	<0.001 ***
	spectral	Maximum frequency	42.64	1.72	43.11	1.72	0.007 **
		Wavelet energy (scale = 5)	0.55	1.08	0.63	1.11	0.020 *
		Wavelet energy (scale = 8)	0.65	1.27	2.27	1.32	0.020 *
		Wavelet entropy	2.11	0.02	2.11	0.02	<0.001 ***
		Wavelet variance (scale = 5)	1.47	3.81	1.61	3.59	0.020 *
		Wavelet variance (scale = 8)	2.03	5.20	2.27	5.16	0.030 *
Gaze		horizontal saccade percentage	0.39	0.13	0.36	0.14	0.007 **
SVD	statistical	Entropy	0.71	0.12	0.78	0.07	<0.001 ***
		Kurtosis	0.91	2.43	0.52	1.51	0.003 **
		Max	0.71	0.09	0.72	0.08	0.007 **
		Standard deviation	0.07	0.01	0.07	0.01	0.001 **
	spectral	Max power spectrum	1.18	0.72	1.01	0.56	<0.001 ***
		Maximum frequency	41.29	3.86	42.35	3.00	<0.001 ***
		Spectral decrease	−5.71	2.12	−5.98	1.96	<0.001 ***
		Spectral kurtosis	4.31	2.25	4.16	1.59	0.003 **

**Table 3:** Descriptions of 5 baseline datasets (EAR-stats, EAR-spectral, Gaze, EAR-stats + Gaze, EAR-spectral + Gaze) and 2 proposed datasets (SVD-stats, SVD-spectral).

Datasets	Description
EAR-spectral	Dataset consisting of EAR-based spectral features
Gaze	Dataset consisting of Gaze-based features
EAR-stats + Gaze	Dataset consisting of EAR-based statistical and Gaze-based features
EAR-spectral + Gaze	Dataset consisting of EAR-based spectral and Gaze-based features
SVD-stats	Dataset consisting of SVD-based statistical features
SVD-spectral	Dataset consisting of SVD-based spectral features

**Table 4:** Accuracy, F1, AUROC performance of each model, corresponding to each dataset of 20-s probing window with stratified 5-fold cross-validation. Stars (*) denote the results obtained by baseline methods in a state-of-the-art webcam-based mind-wandering prediction study [6]. Highest F1 and AUROC are highlighted in bold.

Features	Model	Focused		Not-Focused		AUROC
		**Accuracy**	**F1**	**Accuracy**	**F1**	
EAR-stats *	XGBoost	0.84 ± 0.03	0.79 ± 0.02	0.17 ± 0.04	0.21 ± 0.04	0.53 ± 0.03
	DNN	0.81 ± 0.02	0.77 ± 0.01	0.12 ± 0.03	0.19 ± 0.02	0.49 ± 0.01
EAR-spectral	XGBoost	0.80 ± 0.04	0.77 ± 0.02	0.16 ± 0.07	0.18 ± 0.07	0.53 ± 0.02
	DNN	0.76 ± 0.03	0.75 ± 0.02	0.14 ± 0.04	0.16 ± 0.02	0.45 ± 0.01
Gaze *	XGBoost	0.79 ± 0.03	0.76 ± 0.03	0.18 ± 0.08	0.19 ± 0.08	0.54 ± 0.03
	DNN	0.68 ± 0.02	0.61 ± 0.02	0.12 ±0.03	0.15 ± 0.04	0.50 ± 0.02
EAR-stats + Gaze *	XGBoost	0.83 ± 0.05	0.78 ± 0.04	0.20 ± 0.05	0.23 ± 0.08	0.55 ± 0.01
	DNN	0.77 ± 0.03	0.69 ± 0.02	0.22 ± 0.03	0.24 ± 0.03	0.51 ± 0.02
EAR-spectral + Gaze	XGBoost	0.85 ± 0.04	0.80 ± 0.02	0.19 ± 0.07	0.24 ± 0.08	0.56 ± 0.03
	DNN	0.71 ± 0.02	0.69 ± 0.04	0.28 ± 0.05	0.27 ± 0.03	0.53 ± 0.02
SVD-stats	XGBoost	0.81 ± 0.04	0.78 ± 0.03	0.22 ± 0.08	0.25 ± 0.06	0.52 ± 0.03
	DNN	0.76 ± 0.03	0.76 ± 0.05	0.26 ± 0.04	0.24 ± 0.02	0.49 ± 0.03
SVD-spectral	XGBoost	0.84 ± 0.04	**0.80 ± 0.03**	0.27 ± 0.09	**0.30 ± 0.09**	**0.57 ± 0.04**
	DNN	0.79 ± 0.05	0.78 ± 0.02	0.28 ± 0.03	0.26 ± 0.07	0.51 ± 0.02

## Data Availability

The underlying data supporting Figure 1, Figure 6, Figure 7 and Figure 8, Table 2 and Table 4 were taken from the PAFE dataset, whose information can be found in this record: https://api.semanticscholar.org/CorpusID:251019722 (accessed on 1 April 2024). Please be aware that as the dataset contains privacy-sensitive data, the authors now offer the dataset access primarily for academic research purposes. You may still request access if you wish to use the dataset for purposes other than academic research. In that case, however, your request can be subjected to a more rigorous review, and the authors may ask for additional information. For the same reason, they will not consider requests submitted under personal email addresses such as Gmail. This is to protect the privacy of participants who contributed to our dataset, so please submit a request with a verifiable academic or company email address. Also, providing detailed information about yourself and your purpose in requesting their dataset will help them decide whether to grant or deny access. Inquiries regarding access to the data or additional information should be directed to the first author via email (taekyung@kaist.ac.kr).
